# Stimulus-Induced Motor Afterdischarges in CASPR2 (Contactin-Associated Protein-Like 2)-Positive Peripheral Nerve Hyperexcitability Syndrome

**DOI:** 10.7759/cureus.45643

**Published:** 2023-09-20

**Authors:** Kiren G Koshy, Thomas Iype, Praveen Panicker

**Affiliations:** 1 Neurology, Medical College Trivandrum, Trivandrum, IND

**Keywords:** nerve conduction studies, afterdischarges, electromyography, peripheral nerve hyperexcitability, caspr2

## Abstract

Peripheral nerve hyperexcitability is an uncommon but treatable condition in neurology. Voltage-gated potassium channelopathies, especially contactin-associated protein-like 2 (CASPR2) antibody, are commonly implicated. We present the case of a 16-year-old boy with tremulousness of both feet and twitching of muscles all over the body for three months. Examination revealed irregular, arrhythmic, small-amplitude twitching movements of the toes along with fasciculations in both thighs. Nerve conduction studies were within normal limits. F-wave studies showed a prolonged polyphasic large-amplitude discharge following the compound muscle action potential and obscuring the F waves. Electromyography showed extensive myokymic discharges. The serum autoimmune antibody profile showed strong positivity for CASPR2. He started lacosamide as a symptomatic treatment. In view of the good symptomatic response, further immunomodulation was deferred and he remains on follow-up. We present this case to highlight the role of motor afterdischarges as a diagnostic clue to peripheral nerve hyperexcitability and to review the literature on this interesting finding.

## Introduction

Peripheral nerve hyperexcitability (PNH) is a syndrome characterized by overactivity of the motor nerve fibers, resulting in spontaneous muscle activity in the form of spontaneous muscle twitching. PNH syndromes can be broadly divided into primary PNH (isolated PNH) and secondary PNH (associated with neuropathies) [1]. In recent years, autoimmune causes, especially anti-contactin-associated protein-like 2 (CASPR2)-related disease, have been identified in many cases of primary PNH [2]. PNH presents with muscle twitching, cramps and stiffness, along with paresthesia [3]. Some cases of autoimmune PNH can be paraneoplastic, and this association has been described with tumors of the thymus, lung and ovary [4]. As the clinical presentation of this condition can be non-specific and antibody testing can be expensive, electrodiagnostic studies play a major role in the initial diagnosis of PNH. We present a case of anti-CASPR2-related PNH with motor afterdischarges on routine motor nerve conduction studies and review the literature on the electrodiagnosis of this condition.

## Case presentation

**Figure 1:**
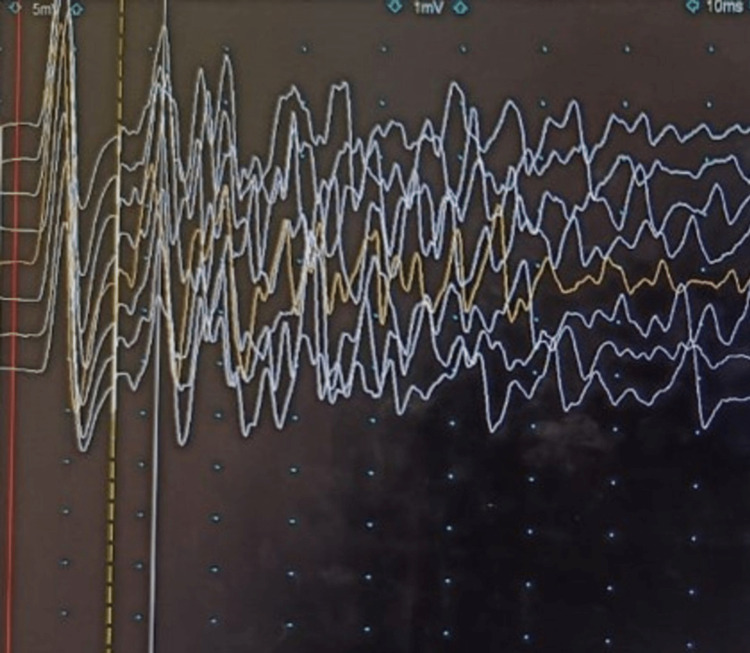
F-wave study in the right tibial nerve showing a prolonged high amplitude polyphasic discharge following the CMAP and obscuring the F waves (sensitivity 1 millivolt per division, sweep speed 10 ms per division) CMAP: compound muscle action potential.

**Figure 2:**
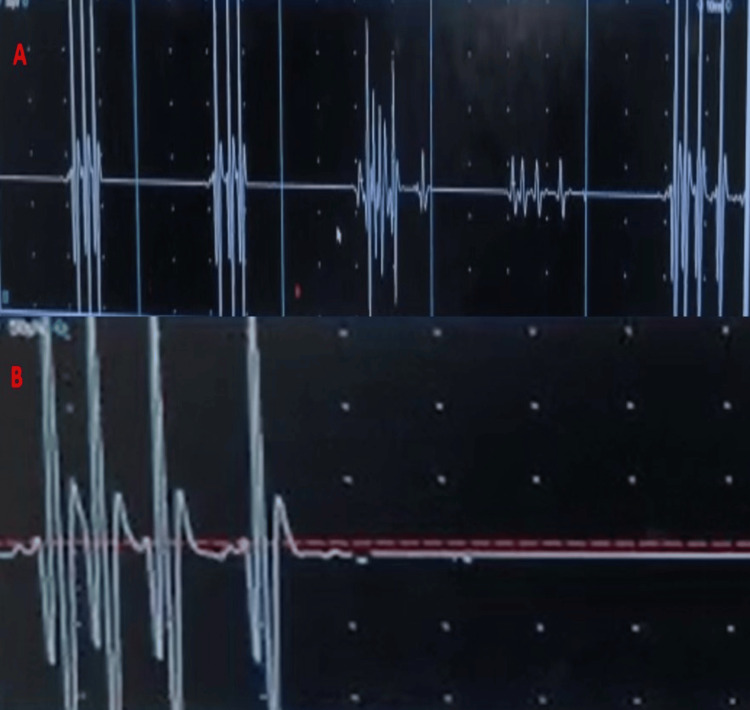
Myokymic discharge A. Needle EMG from the right tibialis anterior muscle showing grouped myokymic discharges. B: Enlarged view of a myokymic discharge. EMG: electromyography.

A 16-year-old student presented with a three-month history of tremulousness on both feet, more at rest. This was accompanied by a vague discomfort in both feet. A month later, he noticed muscle twitching in both thighs, involving both anterior and posterior aspects. Following this, he developed poorly localized pain in both lower limbs. At the time of admission, he reported muscle twitching and pain which were worse at night and were disturbing sleep. There were no systemic symptoms. He had no significant past medical, treatment or family history. Examination revealed irregular, arrhythmic, small-amplitude twitching movements of the toes along with fasciculations in both thighs. There was no significant muscle wasting, and the muscle power and the deep tendon reflexes were normal in all four limbs. The plantar responses were flexor. Sensory examination and gait were within normal limits. There were no thickened nerves. Routine motor and sensory nerve conduction studies were within normal limits. F-wave studies showed a prolonged polyphasic large-amplitude discharge following the compound muscle action potential and obscuring the F waves (Figure 1). Needle electromyography (EMG) done in the right tibialis anterior and rectus femoris muscles showed extensive myokymic discharges (Figure 2). There was no evidence of neuromyotonia. The motor unit action potential morphology and recruitment were normal. Complete blood count, liver and kidney function tests, thyroid function tests and serum electrolytes (sodium, potassium, calcium and magnesium) were normal. Serum testing for contactin-associated protein-like 2 (CASPR2) antibodies (cell-based assay by indirect immunofluorescence) was positive. Chest X-ray and ultrasound of the abdomen (done as part of a paraneoplastic workup) were normal. A diagnosis of peripheral nerve hyperexcitability due to CASPR2 antibodies was made. He was treated with lacosamide (4 mg per kg) with excellent symptomatic benefit. He remains on close follow-up (six months as of date) and remains on lacosamide with a plan to initiate immunotherapy (steroids or intravenous immunoglobulin) if symptoms recur.

## Discussion

Peripheral nerve hyperexcitability (PNH) is a phenomenon characterized by spontaneous bursts of motor unit discharges which manifests as continuous muscle fiber activity [5]. Clinically, it may present as cramp-fasciculation syndrome, neuromyotonia or Morvan’s syndrome. While neuromyotonia is the classic electromyographic finding in PNH, myokymic discharges may be seen in some cases and the EMG may be normal in others. Stimulus-induced motor afterdischarges have been reported in 76% of patients with peripheral nerve hyperexcitability syndrome and may be a more sensitive and less invasive alternative to needle EMG [6,7]. A large series of patients with PNH showed that only around 10% of them were paraneoplastic, with thymoma and carcinoma thyroid being detected on follow-up [5]. The common cause of PNH is autoimmunity to voltage-gated potassium channels, especially CASPR2. Immunotherapy with intravenous immunoglobulin, plasmapheresis and/or steroids has been shown to result in remission in up to 90% of cases [8]. Sodium channel blockers like carbamazepine are also reported to alleviate symptoms [2]. Reversal of the motor afterdischarges has been reported after immunotherapy [9]. A case-control study reports that motor afterdischarges are more consistently seen in PNH when compared to EMG evidence of myokymia, myotonia or fasciculations [10]. Lacosamide has been reported to give excellent symptomatic relief in peripheral nerve hyperexcitability [11]. The maximum yield of motor afterdischarges is obtained from the tibial nerve [7]. Afterdischarges are often best appreciated with a sensitivity of 500 microvolt to 1 millivolt per division which is the usual setting used for the demonstration of F waves. While routine motor conduction studies are normal in most cases, concomitant axonal neuropathy has been reported in some cases [4]. Generally, peripheral nerve manifestations are more often associated with CASPR2 antibodies and central manifestations with leucine-rich glioma-inactivated (LGI1) antibodies. However, co-occurrence of LGI1 and CASPR2 antibodies has also been reported [12].

## Conclusions

Routine nerve conduction studies are a valuable tool in the initial diagnosis of peripheral nerve hyperexcitability syndromes. The demonstration of motor afterdischarges in motor conduction studies is a relatively non-invasive method which can supplement electromyography in the diagnosis of peripheral nerve hyperexcitability syndromes. The combination of clinical suspicion, motor afterdischarges and myokymia or neuromyotonia on electromyography can give a strong clue to the diagnosis of this eminently treatable condition even before specific antibody titers are available.
